# Identifying and addressing data asymmetries so as to enable (better) science

**DOI:** 10.3389/fdata.2022.888384

**Published:** 2022-07-18

**Authors:** Stefaan Verhulst, Andrew Young

**Affiliations:** The Governance Lab, An Action Research Centre at New York University's Tandon School of Engineering, New York, NY, United States

**Keywords:** data asymmetry, data stewardship, data collaboration, Findable, Accessible, Interoperable, and Reusable (FAIR) principles, open data, open science

## Abstract

As a society, we need to become more sophisticated in assessing and addressing data asymmetries—and their resulting political and economic power inequalities—particularly in the realm of open science, research, and development. This article seeks to start filling the analytical gap regarding data asymmetries globally, with a specific focus on the asymmetrical availability of privately-held data for open science, and a look at current efforts to address these data asymmetries. It provides a taxonomy of asymmetries, as well as both their societal and institutional impacts. Moreover, this contribution outlines a set of solutions that could provide a toolbox for open science practitioners and data demand-side actors that stand to benefit from increased access to data. The concept of data liquidity (and portability) is explored at length in connection with efforts to generate an ecosystem of responsible data exchanges. We also examine how data holders and demand-side actors are experimenting with new and emerging operational models and governance frameworks for purpose-driven, cross-sector data collaboratives that connect previously siloed datasets. Key solutions discussed include professionalizing and re-imagining data steward roles and functions (i.e., individuals or groups who are tasked with managing data and their ethical and responsible reuse within organizations). We present these solutions through case studies on notable efforts to address science data asymmetries. We examine these cases using a repurposable analytical framework that could inform future research. We conclude with recommended actions that could support the creation of an evidence base on work to address data asymmetries and unlock the public value of greater science data liquidity and responsible reuse.

## Introduction

The maxim “knowledge is power” is more relevant today than ever before. The data age has redefined the notion of knowledge (as well as power), leading to a greater reliance on access to data and new forms of analysis, such as artificial intelligence (AI) and machine learning (ML). Data access increasingly determines scientific discoveries and advancements. Despite years of progress in implementing open data and “Findable, Accessible, Interoperable, and Reusable” (FAIR) principles (Wilkinson et al., 2016), data asymmetries are a growing problem. If left unaddressed, they can undermine scientific progress and exacerbate existing power imbalances. Below, we argue that we need to become more sophisticated in addressing data (and in turn, power) asymmetries—and their resulting political and economic inequalities—including notably in the realm of open science, research, and development.

## Data asymmetries

In 2016, a group of researchers published the “FAIR Guiding Principles for scientific data management and stewardship” in *Scientific Data* (Wilkinson et al., 2016). The authors intended their piece to provide guidelines to improve the findability, accessibility, interoperability, and reuse of digital assets. These principles have had resonance worldwide. However, there remains a large divide or disparity in access to data, which we call *data asymmetries* (Dodds, 2017). Even when disparities are overcome, there often exist pervasive inequalities to the extent in which individuals and groups can *actually* benefit by deriving new insights or informing innovation from their functional access to information. In short, stakeholders differ in their abilities to access and translate data into insight and actionable intelligence.

The nature of this divide can take many forms depending on the relationship between data suppliers and demand-side actors that could derive value from access and reuse of data. To date, we have documented the following types of data asymmetries:

- **Business-to-consumer (B2C)**: B2C asymmetries dominate much of the current public discussion. Such asymmetries have grown increasingly common with the datafication of consumption patterns and typically occur when companies collect data on their users while providing services or selling goods. For example, companies might collect data related to transaction or browsing histories, or a variety of socio-demographic markers. As a result, companies often possess a disproportionate amount of data on their users—information that users may not even be aware of having surrendered.
- **Business-to-business (B2B)**: Recent years have also witnessed the emergence of a number of large data monopolies that dominate their sectors and the broader economy—so-called B2B asymmetries. These companies have access to huge amounts of data collected and processed across various domains (e.g., search data, location and mobile phone data, and consumer spending data), and their ability to combine this data or train ML algorithms to derive insights from the information results in de facto barriers to entry. There are concerns that B2B data asymmetries may stifle innovation and competition, as well as hurt the rights of consumers, leading to calls for greater regulation and better enforcement of antitrust law, perhaps extending so far as to the breakup of some of these large players (Srinivasan, 2021).
- **Business-to-government (B2G)**: Policymakers are increasingly turning their focus on data asymmetries in the B2G space, which refers to the ability of governments to access important datasets that companies might hold. Influential actors, including the High-Level Expert Group to the European Commission on B2G Data Sharing, are writing on the ways government decision-making and service delivery can be hampered by a lack of access to data and insights that are held in the private sector and, at present, solely used for commercial purposes (European Commission Directorate-General for Communications Networks Content Technology, 2021). For instance, the High-Level Expert group notes that “due to organizational, technical and legal obstacles […] data-sharing partnerships are still largely isolated, short-term collaborations (European Commission Directorate-General for Communications Networks Content Technology, 2021).”
- **Government-to-citizen (G2C)**: The open data movement, which grew out of freedom of information laws and flourished as part of global moves toward open source and Web 2.0 approaches, aims to address government-to-citizen (G2C) data asymmetries (Verhulst et al., 2020). Its premise is that data collected by the government (and funded by taxpayers) are often siloed and hoarded, limiting transparency and the capacity of citizens to derive value from it (Verhulst and Young, 2016). In 2012, New York City passed the Open Data Law, requiring city agencies publish data on a single, publicly accessible data portal. The data from this portal has allowed city residents to make city agencies accountable, launch new businesses, and otherwise understand the communities in which they live and work (NYC Open Data, 2022).
- **Business-to-science (B2S)**: The open data field has paid comparatively little attention to another important data asymmetry slowing societal progress and advancement—B2S data asymmetries. As is the case across domains, the private sector holds massive amounts of data that could provide value for scientific inquiry and research across disciplines. Too often, that information remains siloed due to businesses' concerns regarding competitive advantage and trade secrets, privacy harms, or security risks. It can also be siloed because of researchers' lack of recognition of the types of valuable datasets held in the private sector that could support their work and a belief that only data generated in a lab can truly enable new scientific insight. These challenges, as well as the relative lack of systematic, repeatable operational and governance models to enable B2S data collaboration, lead to persistent transaction costs for the scientific community related to finding, extracting, formatting, and integrating data to support their analyses (Mons et al., 2020). It can also lead to untapped opportunities for society as achievable, potentially transformative scientific insights continue to go unrealized.

These data asymmetries can also relate to and be compounded by other asymmetries of power and influence, including notably between stakeholders in the Global North and Global South (Abimbola et al., 2021).

This taxonomy of data asymmetries can be further nuanced by examining different types of data or “digital objects.” The specific types of assets that could be stewarded in the public interest and the dominant asymmetries are domain-specific. This exploration is out of scope for this initial contribution, but future work could add greater depth (da Silva Santos, 2021).

## Value proposition of addressing data asymmetries for open science through access to private sector data

In many ways, a move toward more consistent B2S data collaboration is aligned with core tenants of the open science movement, namely a shift in the prime focus of research turning away from publishing and “toward knowledge sharing (Burgelman et al., 2019).” There is a wide and growing body of evidence that demonstrates the public and scientific value of the reuse of data collected for one purpose by actors working to create new insights and public value (Verhulst, 2019). Yet the supply of and demand for data and data expertise are currently widely dispersed across government, the private sector, and civil society and most often poorly matched. There is a need to make data more accessible and more affordable for a wider variety of stakeholders.

Indeed, to achieve the goals of open science, stakeholders across sectors need to contribute to a more robust and dynamic data ecosystem. This ecosystem and infrastructure will, by necessity, “involve a mix of players, including commercial and public ones,” not just traditional scientific researchers or data providers (Mons et al., 2017). It is also important to note that the collaborative use of private sector data assets, as opposed to data being made fully open access, does not contravene the FAIR principles. As Mons et al. (2020) notes, “FAIR is not equal to open: The ‘A' in FAIR stands for accessible under well-defined conditions, while reusability conditions are covered in the requirement to have a clear, machine-readable license as per the R of FAIR (Mons et al., 2020).”

Open data approaches have failed to generate the envisioned impact thus far. Given the realities of our current data age, actors working in the public interest will likely need to engage with private sector data holders to generate lasting and meaningful change. More collaborative approaches, such as those highlighted in Table 1, are urgently required.

**Table 1:** Overview of types of data asymmetries.

**Data asymmetry**	**Notable manifestation**
Business-to-consumer (B2C)	Companies possess a disproportionate amount of data on their users—information that users may not even be aware of having surrendered.
Business-to-business (B2B)	Large data monopolies can dominate sectors and the broader economy, limiting other businesses' capacity to access and use data.
Business-to-government (B2G)	Government decision-making and service delivery can be hampered by a lack of access to data and insights that are held in the private sector and solely used for commercial purposes.
Government to Citizen (G2C)	Data collected by the government (and funded by taxpayers) are often siloed and hoarded, limiting transparency and the capacity of citizens to derive value from it.
Business-to-science (B2S)	The private sector holds massive amounts of data that could provide value for scientific inquiry and research across disciplines, yet that information remains siloed due to businesses' concerns regarding competitive advantage and trade secrets, privacy harms, or security risks.

## Private sector data portability and liquidity for open science

One of the approaches that have been considered as a means to counter data asymmetries is increased data liquidity. Having gained traction in the past few years, *data liquidity* refers to “the ease of data asset reuse and recombination (Rodriguez et al., 2021).” Unlike capital assets, for which the value tends to deteriorate over time, data's value does not decrease and can, on the contrary, increase, as the data are reused and recombined in different ways. As a consequence, the easier the reuse and recombination of the data, the higher its liquidity.

Liquid data can serve several purposes, from being monetized, enabling innovation, contributing to research, or helping institutions resolve some of their most pressing issues. Most data, however, continue to stagnate in silos, increasing the likelihood of them being incomplete, wrongly classified, and inaccessible to use by their subjects and by others (European Commission Directorate-General for Communications Networks Content Technology, 2021).

In recent years, the idea of data liquidity has garnered increased attention and gained traction in the healthcare industry. The Mayo Clinic, for example, announced a ten-year partnership with Google in 2019 to advance its health system's cloud-based AI and ML capabilities to improve its data liquidity (Miliard, 2019). Pushed by the COVID-19 pandemic, many interoperability and data liquidity conversations have been centered on making data use and exchange more fluid for the greater good of patients, healthcare providers, and researchers.

Academics have called for better reuse of data in the private sector, where most of the data reside. Researchers at MIT have also introduced the concept of *strategic digital data assets*, arguing that data should be reused and monetized to best achieve data liquidity (Rodriguez et al., 2021). Several hurdles remain, however, to operationalizing data liquidity for science. One of the biggest challenges is the perception of data as a company asset leading to anti-competitive concerns. As a result, many companies are reluctant to share their data and let them flow freely. Because a lot of the world's data are in the hands of private sector companies, this remains a considerable issue. Different operational models based around differing conceptions of access and use have emerged (see Table 2).

**Table 2:** Emerging models of addressing B2S data asymmetries.

**Operational model of data stewardship for open science**	**Examples**
**Enabling Independent Uses of Data**	
Research data portals	Microsoft Research Open Data
Open science data commons and marketplaces	European Open Science Cloud
**Enabling Cooperative Uses of Data**	
Research partnerships and consortia	Cuebiq Data4Good
Brokerages and intermediaries	Social Science One
**Enabling Approved Uses of Data**	
Research passports	Global Alliance for Genomics and Health (GA4GH)
Data safe havens and distributed analytics	Canadian Institute for Military and Veteran Health Research Data Safe Haven (CIMVHR)

Another issue is the interoperability of data. *Data interoperability* “addresses the ability of systems and services that create, exchange and consume data to have clear, shared expectations for the contents, context and meaning of that data (Castro, 2021).” Without data interoperability, efforts to promote data liquidity will remain ineffective.

*Data portability* may offer solutions to some of these problems (Verhulst and McMurren, 2020). Users of online and mobile applications generate valuable data through their use of digital services. These data grow through their digital lives to accumulate and be considered as their “digital capital (Exposito-Rosso et al., 2021).” Data portability gained prominence as a concept in 2018 through the European Union's General Data Protection Regulation (GDPR) and the California Consumer Privacy Act (CCPA). Data portability's purpose is to protect users from having their data stored in “silos” or “walled gardens” that are incompatible with one another. The right to data portability refers to “the ability of users to obtain and transfer a copy of their data from one data controller (e.g., an app or online service) to another (Castro, 2021).”

Unlike many restrictive consumer protection laws, data portability is a permissive provision intended to increase data use and sharing (Castro, 2021). The concept of data portability promises to give consumers more power over their data while easing restrictions on data flows. By doing so, it places the data back into the user's hands and out of the silos, making it more readily available for reuse and recombination by multiple organizations. These reuse and recombinations can be facilitated by data stewards, responsible data leaders empowered to seek new ways to create public value through cross-sector data collaboration (see Table 3).

**Table 3:** Functions of re-imagined data stewards.

**Re-imagined data steward functions**	**Description**
1. Stewarding data assets for and in the public interest: data audit, assessment, and governance	Determining and assessing the value, potential, and risk of data held within an organization.
2. Stewarding relationships: partnership and community engagement:	Proactively and responsively reaching out to and vetting potential partners or data users.
3. Stewarding internal resources, expertise, and authorities: internal coordination and data ops	Securing internal coordination and establishing data operations.
4. Stewarding sustainability: nurturing data collaboratives to sustainability	Gathering the needed resources and support to ensure broad and long-term impact.
5. Stewarding insights: dissemination and communication of findings	Raising awareness, disseminating findings, and communicating outcomes to the public and relevant stakeholders.

The concept of data portability, as introduced through the GDPR includes requirements for common technical standards (Wikimedia Foundation, 2022) “to facilitate the transfer from one data controller to another, thus promoting interoperability (Thomas, 2010)”. Article 20 of the GDPR states that individuals can obtain their information “in a structured, commonly used and machine-readable format” and use data that a company holds on them. Given its presence in the European supranational legislation, much of the research around data portability and its uses and advantages have focused on Europe. Some of the latest research has focused on how data portability will influence the private sector by stimulating competitiveness and enabling the development of new businesses (de Streel et al., 2021). The concept could, however, be adapted to respond to the data asymmetries affecting various fields that have been listed above. For example, launched in 2018, The Data Transfer Project (Data Transfer Project, 2022) demonstrates an industry-led effort to promote the transfer of data between partner platforms.

## Emerging models and brief case studies

This section provides a series of brief case studies on notable efforts to address science data asymmetries^1^ Across all of these models and approaches, the FAIR Principles provide important guidelines and signposts. As Mons et al. (2017) state:

[T]here is a need for a set of community-acceptable “rules of engagement,” that define how the resources within that community will/should function and promulgate themselves. These rules of engagement may vary depending on the needs or constraints within any given community, but in each case, the FAIR guidelines assist the interaction between those who want to use community resources and those who provide them. FAIR guiding principles provide a scaffold for building such rules of engagement within each community (Mons et al., 2017).

## Enabling independent uses of data

On the most open end of the spectrum, several models enable independent uses of previously siloed data. These approaches often involve *data portals, platforms*, and *products* that make datasets available for download or enable users to manipulate and analyze data online. These approaches generally do not require users to demonstrate any particular capacity or credentials, and use cases are not subject to approval by the data providers.

These approaches are often most appropriate for data that do not contain significant sensitivity from a privacy, data protection, or competitive advantage perspective. Data assets made accessible through these approaches can inform research that is reproducible. This section provides several practical examples of this process.

### Research data portals

The most open model for supporting researchers' use of previously siloed data takes the form of open research data portals. As is the case with open government data portals, these platforms provide open access to data, often with no restrictions on the type of user or use case. The data offerings are made accessible as public goods, and are often—but not always—structured in a way that supports machine readability. One such example is the Microsoft Research Open Data initiative, a collection of free datasets from Microsoft Research on natural language processing, computer vision, and other sciences (Microsoft Research, 2022).

#### Microsoft research open data

Microsoft Research Open Data is an initiative by Microsoft Corporation's research subsidiary to make datasets produced by the organization available to other researchers. The public can find the datasets on topics such as natural language processing and computer vision on the Microsoft Research website, and they can download them to an Azure-based Virtual Machine or Data Science Virtual Machine. Like other Microsoft Research products, the resource follows the FAIR principles whenever applicable. The effort seeks to “simplify access to [shared] data sets, facilitate collaboration between researchers using cloud-based resources, and enable the reproducibility of research (Mandava, 2018).”

### Open science data commons and marketplaces

While research data portals are geared toward making previously siloed data available for download by any user, data commons and marketplaces can offer several functionalities and means of engagement. These platforms are generally open to all—though often based on some type of registration process—and seek to provide a more robust and diverse set of capabilities to enable data access, storage, and analysis in the interest of advancing open science. They often commingle datasets from various sources, such as private sector data holders, public bodies, non-governmental organizations, and researchers themselves. More than just an approach for cloud-based storage or data download, they aspire to establish an infrastructure to support activities across the science data value chain.

#### European open science cloud

The European Open Science Cloud (n.d.; EOSC) is a service that aims to enable 1.7 million researchers in Europe to store, share, and reuse data across nations and scientific disciplines through an open science cloud and without leaving their desk (Burgelman et al., 2019).” With support from the European Union's Horizon 2020 research and innovation program, EOSC “mobilizes providers from the EGI Federation, EUDAT CDI, INDIGO-DataCloud and other major European research infrastructures to deliver a common catalog of research data, services and software for research (Manzi, 2020).” More than a data portal or access point, EOSC acts as a hub for news, partnerships, training, publications, research communities, and other services that were previously dispersed across providers or otherwise burdensome to discover and access.

## Enabling cooperative uses of data

*Data cooperatives* see data providers engage directly with users on the demand side and/or intermediaries that help to match supply with demand. These approaches allow data providers to retain greater control over what types of use applications their data can support. These models can support the analysis and reuse of data that would be unfeasible or unlikely to be made fully accessible. The direct cooperation between data providers and researchers (or intermediaries) can help to unlock more sensitive data from either a data protection or commercial standpoint. These models can also be more time- and resource-intensive than efforts that support independent use of data by researchers. The lack of broad accessibility can also create reproducibility challenges, raising questions about the accuracy and representativeness of datasets and eliciting scrutiny over data providers' influence on research findings.

### Research partnerships and consortia

Private sector data holders may forge direct partnerships with researchers to enable the reuse of data for scientific purposes. Whether one-to-one or one-to-several, these partnerships or consortia tend to be “high-touch,” with data holders working closely with researchers. Data holders generally play a role in identifying and approving researchers and use cases and provide support throughout the data life cycle and science value chain. These efforts often rely on data-sharing agreements or other contracts.

#### Cuebiq Data4Good

Cuebiq, for example, is a location intelligence company that has been sharing its data assets with partnering research organizations through the Data for Good Initiative (Cuebiq, n.d.). Since its launch in 2017, the project has provided researchers with anonymized data on location patterns, which has enabled geospatial research such as MIT Media Lab's Atlas of Inequality (“The Atlas of Inequality”) and data-informed responses during the COVID-19 pandemic (MIT Media Lab, n.d.). Cuebiq has institutionalized roles and functions for supporting research by providing functional access to its location intelligence data through, for example, the existence of a Director of Research Partnership and Data for Good (Winowatan et al., 2020).

### Brokerages and intermediaries

Cooperative efforts to address data asymmetries to support science do not always involve bilateral relationships that match supply with demand. Some initiatives involve trusted third parties that play a role in brokering connections between supply-side data holders and researchers on the demand side. These intermediaries might also provide additional expertise or value, such as additional governance capacity, processing or analytical rigor, or legal grounding. Brokerages and intermediaries can ease the burden of transaction costs facing data holders, potentially paving the way for more contributions to scientific efforts.

#### Social science one

Social Science One is a research consortium hosted by Harvard's Institute for Quantitative Social Science that was co-founded with Meta (formerly Facebook; Harvard's Institute for Quantitative Social Science, n.d.). Social Science One has faced numerous obstacles since its initial conception, yet it provides an instructive example of an intermediary that connects social scientists with private sector data. In the initial stage of its partnership with Facebook—now Meta—to explore social media's impact on democracy, both technical and legal challenges limited researchers' access to useful data in a timely manner, especially as Meta announced its intention to manipulate data to protect personal information, a method which could introduce significant biases (Pasternack, 2019). Beyond the frustration caused by the downgraded granularity of shared data, Meta also acknowledged that datasets that had already been explored by the researchers included a serious error—an accidental exclusion of data on U.S. users without a detectable political affiliation. This flawed data, which lacked information for almost half of Meta's users in the country, had already been shared with at least 110 researchers and required extensive reexamination upon the receipt of corrected data (Timberg, 2021). This example demonstrates the need for external regulations for social media companies to ensure responsible, secure, and transparent data sharing mechanisms that adequately cater to social science research.

## Enabling approved uses of data

Several efforts are underway to stake out a middle ground between fully open uses of previously siloed data and more cooperative engagements that could involve significant transaction and coordination costs. These efforts seek to support external uses of data held by private sector businesses or other institutions. While these efforts do not involve direct cooperation or collaboration between supply-side actors and science researchers representing the demand, processes and procedures are in place to ensure that data are only made accessible for approved use cases. This middle ground can establish confidence among data holders that their information will not be used for nefarious or inappropriate reasons, while also increasing accountability for both supply- and demand-side actors. Researchers can be held accountable for unapproved uses of data, and suppliers may face public or industry scrutiny if data are not provided to researchers that satisfy all predetermined criteria.

### Research passports

Research passports are a mechanism through which researchers can gain access to data held across one or more silos. Upon demonstrating their credibility and that they meet certain criteria or requirements, researchers gain a digital identity token or artifact that unlocks their access to data streams that are not openly accessible. Each research passport involves different criteria—though more cross-cutting solutions could be established. These passports, or “visas” in some instances, provide a “technical representation of data governance process outcomes with a durable assurance that the person(s) accessing the data [has] been authorized to do so (Voisin et al., 2021).”

#### Global alliance for genomics and health (GA4GH)

The Global Alliance for Genomics and Health (GA4GH) “is a policy-framing and technical standards-setting organization, seeking to enable responsible genomic data sharing within a human rights framework (Global Alliance for Genomics Health, 2022).” The organization is hosted by the Broad Institute of MIT, the Harvard and Ontario Institutes for Cancer Research, Wellcome Sanger Institute, and European Molecular Biology Laboratory (EMBL)”s European Bioinformatics Institute with support from Genome Canada, the Canadian Institutes of Health Research, the National Institutes of Health, the Wellcome Sanger Institute, the Medical Research Council, and the National Institute for Health Research. GA4GH provides individual researchers with a passport in the form of a “machine-readable digital identity that conveys roles and data access permissions (Voisin et al., 2021).” These roles and data access permissions, called “visas,” are created and distributed by data access committees and other data stewards responsible for administering databases compiling biomedical data.

### Data safe havens and distributed analytics

Data safe havens are processing environments that allow researchers to access, store, and analyze data securely and without having the capability of extracting raw data. This model enables research on proprietary or sensitive datasets and allows researchers to generate and extract findings to support their work, all while guarding against unauthorized access or breaches. Suver et al. (2020) describe a data safe haven as a “collective resource kept in a secure computing environment and managed with appropriate ethical and legal governance for the mutual benefit of individuals, communities, and the society (Suver et al., 2020).” These safe havens often make accessible clones of synthetic datasets, rather than the raw or original datastreams, to add an additional layer of security beyond access and extraction controls.

Distributed analytics refer to a related approach by which researchers “bring the model to the data (Hardjono et al., 2019)” and apply their analyses or algorithms on siloed data sets either in a data safe haven or on the business server on which those data are natively housed.

Data safe havens and distributed analytics efforts can be more practicable for private sector actors than direct data sharing for two reasons. First, only approved users can analyze data for approved use cases. Second, the results or findings of the analyses are permitted to leave the safe haven or analytical environment, not the data themselves. The latter can be seen as a beneficial feature for unlocking private sector data for scientific analysis and augmenting B2S practices. However, it can also be seen as a “bug” or disabler of downstream openness or reproducibility of insights derived from the work.

Not only do these serve as a solution to privacy, security, and competitive disincentives faced by data-holding businesses, but also provides a means for navigating complex and often opaque regulations impacting data-sharing within and across different jurisdictions and national borders (Suver et al., 2020).

#### Canadian institute for military and veteran health research data safe haven (CIMVHR)

The CIMVHR Data Safe Haven (CDSH; Martin et al., 2021) was established to provide a “secure repository and analytics platform for data acquired and held by individual research projects and their affiliated organizations or institutions” to advance the needs and interests of active duty Canadian military, veterans, and their families (Martin et al., 2021). The safe haven is administered by the Queen's University Center for Advanced Computing and provides a secure data processing environment where researchers can analyze military members' and veterans' health data. Projects and new data intake are subject to approval by project organizers. Each approved project or use case is provided with a unique workspace in which researchers can conduct their analyses on aggregated datasets. Data transfers between safe and secure workspaces within the safe haven must be authorized by project leads. In addition to security measures, the safe haven provides data accuracy verification and robust computing resources and capacity.

## Key enabler: Data stewardship

One of the major challenges in the data ecosystem is this relative lack of capacity and layer of human infrastructure that is empowered to forge collaborations and support public-interest uses of private sector data. This is true across sectors, not just in the scientific and data community. The problem is likely to grow more acute as data, data science, and data protection legislation grow increasingly complex.

As it stands, most private sector data holders (as well as civil society actors and public sector entities) do not have clearly defined roles and responsibilities aimed at increasing the cross-sector flow of data to solve public problems, improving decision-making in the public interest, and generating new scientific insights. There currently exists a largely unmet need for professionalizing the supply of already collected data to enable more effective and systematic data provision and collaboration that benefits the public good.

At the broadest level, the responsibility of a data steward is to ensure end-to-end responsibility for the collection, storage, handling, and usage of data. This responsibility can be divided into three categories. First, data stewards must collaborate responsibly to unlock data when there is a public interest case. Second, they must protect stakeholders by managing data ethically to prevent harm and misuse. And finally, they must take action to ensure that insight is shared and translated into meaningful impact.

Data stewards do not represent an entirely new profession. Rather, their role could be understood as an extension and re-definition of existing organizational positions that manage and interact with data. Traditionally, the role of a data officer was limited either to data integrity or the narrow context of internal data governance and management, with a strong emphasis on technical competencies. This narrow conception is no longer sufficient, especially given the proliferation of data and the increasing potential of data sharing and collaboration. As such, we call for a re-imagination of data stewardship to encompass a wider range of functions and responsibilities, directed at leveraging data assets toward addressing societal challenges and improving people's lives.

Mons et al. (2020) define data stewardship as “treating data and the associated research objects with the utmost care, with the aim to make them reusable for discovery as long as they are valid (Mons et al., 2020).” Our definition is related but somewhat distinct. In this paper and elsewhere, we define data stewardship as “functions and competencies to enable access to and reuse of data for public benefit in a systematic, sustainable, and responsible way (Verhulst, 2021).”

There exists an increasing recognition that for data stewardship and sharing to create public and scientific value to achieve success, “it must be a truly multi-professional endeavor,” equipped with a human infrastructure that possesses the mandate and capacity to “create spaces for different types of data to be curated, shared, discoverable, and reusable in an ethical and timely way (Woods and Pinfield, 2021).”

Establishing this human infrastructure lies “at the very basis of the needed revolution in scientific methods” as the move toward more open and effective science in the data era faces “many more socio-cultural hurdles… than technical ones (Mons et al., 2020).” A landscape analysis of open science studies performed by Woods and Pinfield (2022) found that “disciplinary data champions [need] to model good practice and drive cultural change (Woods and Pinfield, 2021).”

In the private sector, embracing data stewardship to enable better science will require the upending of a “stubborn” commitment to the idea that “only peer reviewed literature and curated databases are credible sources of scientific legacy information (Mons et al., 2020).” Indeed, as stated by Mons et al. (2020), “dogmatism about how data should be handled is an impediment to scientific progress (Mons et al., 2020).”

The roles and functions of data stewardship can also be served by “honest brokers” positioned to match the supply of data held in the private sector with the demand for it in the scientific community (Suver et al., 2020).

Proponents of this emerging form of data stewardship can look to other data or information-focused roles and functions that have taken hold over recent decades. Chief Information Officers (CIOs), for example, became mainstream in the mid-1980's as businesses saw the strategic importance of information technology to their financial success. A 2016 study identified five key reasons that CIOs have historically struggled to become established: “(1) misunderstanding the transition, (2) ambiguity in defining IT success, (3) ambiguity in role expectations, (4) poor relationship management with peers, and (5) pushing change at the wrong pace (Gerth and Peppard, 2016).”

In 1994, a landmark study of corporate privacy policies found the privacy arena was defined by a lack of attention. Executives did not consider it a strategic issue (Smith, 1994). They left decisions to mid-level managers with little relevant expertise. From the late 1990's to 2000's, this situation changed. Companies in the financial and health sectors created Chief Privacy Officer (CPO) positions to respond to these concerns. Today, the International Association of Privacy Professionals reports 23,000 members. A combination of regulatory activities—including those initiated by the FTC—advocacy efforts from experts and stakeholders, and a public pressure and negative media attention falling on privacy breaches helped to catalyze and incentivize businesses to enshrine the role (Bamberger and Mulligan, 2010).

Ultimately, data stewards are professionals empowered to create public value by reusing data and data expertise, identifying opportunities for productive cross-sector collaboration, and proactively requesting or enabling functional access to data, insights, and expertise. Data stewards are active in both the public and private sectors, promoting trust within and outside their organizations. They are essential to data collaboratives—including but not limited to data collaboration for science—by providing functional access to unlock the potential of siloed data sets. Data stewards represent a new and increasingly essential link in the data value chain for science and beyond.

## Functions and competencies of re-imagined data stewards

Central to these efforts is our ongoing initiative to define the roles of data stewards and encourage the emergence of new data-driven professionals. Our research in this space—including the curation of global repositories containing hundreds of data collaboratives (The GovLab, 2017) and data partnerships to address COVID-19 (The GovLab, 2020)—and engagement with a growing data stewardship community of practice has indicated that these professionals generally serve three key roles.

The emergence of data stewards in businesses and institutions follows other notable data, knowledge, and information roles established. Chief data officers, chief privacy officers, and chief knowledge officers, to name a few, serve related but distinct roles to how we define data stewards, and similar to these other professions can be located within organizations in different places be it the executive office, the policy or business department and so on, depending on the particular organizational chart of the respective organization.

First, data stewards drive collaboration. They have a responsibility and mandate to unlock data when there is a public interest case—including the potential to generate new scientific insight by providing researchers with functional access to data. Second, they have a responsibility to protect data by managing datasets they hold—and make accessible—in an ethical manner that proactively prevents harm and misuse. Third, they have a responsibility to act upon opportunities to create public value and scientific insight by ensuring insights are shared with relevant actors and positioned for meaningful use and action.

More specifically, data stewards serve several functions and demonstrate a set of competencies to provide functional access to previously siloed data and create new public value. First, they lead data audit, assessment, and governance. Data stewards monitor and assess the value, potential, and risk of all data held within their business. Doing so involves formulating and determining domains where the data they hold could contribute meaningful value and insight; scoping and iterating assessments of “minimum viable” data needed for a particular purpose or research question; identifying and documenting assets held internally; considering the ethical and fundamental rights implications and other risks of providing functional access to datasets for scientific use cases; and helping establish operational, technical and governance models to validate ways to measure impact.

Second, data stewards drive partnership and engagement with relevant stakeholders and communities. In this role, data stewards forge relationships toward vetting potential partners (or partner profiles). They can also play a role in informing data subjects or impacted communities of the insights generated from new collaborations and scientific advancements. Particularly in more cooperative models, data stewards become a point of contact regarding the reuse of data and work to identify and engage with potential partners and stakeholders. By its nature, this work is user-driven and geared toward engagement with the users of data assets, products and insights. Stewards are often responsible, together with their legal teams and counsel, for shepherding through data-sharing agreements, licensing, and other contractual relationships as necessary.

Third, data stewards steer internal coordination and data operations. In order to unlock the public and scientific value of data they hold, data stewards must guide internal resources, expertise and authorities. This role involves the management of internal relations to gain approval from actors within the organization and coordinate with them to ensure that all stakeholders and organizational leaders are informed and aligned. The steward also engages in helping to establish data operations to map and match internal resources, expertise, and skills in order to enable data collaboration. As mentioned above, different operational models require different types of internal configurations—from establishing and hosting data portals and platforms to creating and administering safe and secure data havens and processing environments.

Fourth, data stewards seek to nurture sustainable data collaboratives.

In this role, data stewards work with internal and external stakeholders to gather necessary resources and support broad, long-term impact and sustainability of their work to enable reuse of data to create public and scientific value. This involves institutionalizing data innovation to make the reuse of data systematic; developing the business case to scale and sustain data innovation; and measuring impact and sharing insights to build a societal and business case for data stewardship.

Finally, data stewards drive the dissemination and communication of findings. Data stewards act as the face of a company's data projects and are responsible for communicating shared outcomes from their work to external actors. In this role, they are stewarding insights, and their responsibility is to raise awareness with users, partners, governments, and other stakeholders that could support greater uptake and reuse of data, and build on a set of emerging good practices in the space. They also communicate with actors on issues such as regulatory compliance and contractual obligations, and help translate data intelligence into decision intelligence.

## Risks and challenges of increased access to private sector data for open science

The value proposition of an increased use of private sector data to address data asymmetries and spur scientific discovery is clear: it bridges the information gap between private, public, and civil society sectors to allow practitioners across the board to contribute to “for good” practices. However, these approaches can introduce certain risks to the principles and objectives of FAIR and open science. We describe a few of these risks and challenges here.

### Inequitable access

Some models for providing researchers access to private sector data are not fully open and thus lead to inequity in access. More cooperative models often rely on direct engagement or pre-existing relationships between demand-side researchers and supply-side data holders. Researchers with potentially impactful lines of inquiry but who lack relationships with data suppliers can face challenges in accessing useful datasets—a demonstration of the type of inequity challenges that open science seeks to address in other contexts.

In addition to these questions of relationships and connections, researchers seeking to use private sector data may face legal obstacles. Data-sharing agreements, licenses and other legal frameworks are often necessary for unlocking access to private sector data for research, potentially creating barriers to entry for some (Woods and Pinfield, 2021).

Finally, researchers could require some sophisticated technology and/or human capital to access, store, analyze, and protect private sector data (Staunton et al., 2021). Especially in cases involving very large datasets, researchers might require a level of computing power that extends beyond what is feasible in their institution. Data providers may require evidence of necessary capacity prior to making datasets available for research purposes.

### Enabling downstream reproducibility and reuse

Research undertaken through distributed analytics or in data safe havens are especially prone to challenges related to evaluation and reproducibility. Since data are not fully open, external parties are unlikely to meaningfully assess the quality and representativeness of data used in scientific analyses and face similar challenges in reproducing findings (Suver et al., 2020).

While these challenges can impact the reproducibility of research, limited openness of data used for research does not necessarily contravene FAIR principles. Mons et al. (2017) note that “FAIR simply describes the qualities or behaviors required of data resources to achieve–possibly incrementally–their optimal discovery and scholarly reuse (Mons et al., 2017).”

To help address reproducibility and reuse challenges, new incentive and performance structures will be needed to open up data, including “data level metrics to credit authors for each reuse, such as downloading, data citations and so on (Woods and Pinfield, 2021).” Such a demonstration of uptake and influence is essential for researchers, but “naming and faming” in this way can also act as a key, and often missing, incentive for private sector engagement in scientific initiatives.

Private sector data stewards might participate in open science efforts for a number of reasons—including corporate social responsibility compliance, soliciting new research insights that could inform novel business models, or retaining internal talent that would be compelled by contributing to state of the art scientific endeavors. Bolstering corporate reputation through the demonstrated value and (re)use of private sector data can also be an important, and often missing, driver of such efforts. This aligns with Burgelman et al. (2019) argument that “changing the reward and incentive system for researchers is a key open science challenge (Burgelman et al., 2019).” Researchers' incentives for contributing to the emerging open science movement will be disrupted and dampened in cases where they do not have the capacity to share important datasets that informed their analysis with the broader community.

### Transaction costs

For each of the models discussed above, data providers must undertake activities such as “selection, composition, curation, and annotation” to enable external reuse for scientific purposes (Suver et al., 2020). Each model may require additional and potentially time- and resource-intensive operations, such as hosting an open science portal or platform; establishing legal agreements to enable cooperative use; and creating and ensuring alignment between training and validation datasets in data safe haven efforts.

Data providers often lack incentives for taking on these transaction costs due to the lack of clearly defined business models for supporting science. Even data repositories that were developed with the specific intention of supporting open science are facing “increasing financial pressures that can undermine their long-term sustainability (OECD Global Science Forum, 2017).” Private sector actors could determine that these financial pressures and sustainability challenges are not worth addressing if doing so does not support some type of business model or revenue stream.

Moreover, distributed analytics approaches can still introduce some level of risk to data providers. The model guards against unauthorized access or data breaches, but data stewards “must also consider the security risks associated with allowing outside code to be run inside their protected computing environment (e.g., tampering; Suver et al., 2020).”

### Data hugging and disincentives to collaboration

To be clear, some barriers to the reuse of private-sector data for science purposes were established intentionally by data holders to protect their valuable assets. Businesses with potentially useful data for research purposes span industrial sectors and their data stewardship objectives are subject to different levels of complexity. While, as described above, models are emerging for “bringing the algorithm to the data” and other minimally invasive analytical techniques, some data holders in the private sector are likely to maintain a defensive posture and seeks ways to build walls around their data assets. Businesses may balk at increased data collaboration due to cybersecurity concerns, risks of data subjects being reidentified in shared datasets, and the potential for data made accessible to being used in unapproved artificial intelligence models. While these risks can emerge, especially in sensitive industries, new insights and strategies for governing and operating a data collaborative safely and legitimately could help those with real or inflated concerns steward their data in the public and scientific interest.

## Recommendation actions and next steps

Data asymmetries remain a growing problem in today's data ecosystem, and, if left unaddressed, could undermine scientific progress and exacerbate existing power asymmetries. With this paper, we seek to fill the current knowledge gap regarding the current state of data asymmetries globally by providing a taxonomy of asymmetries as well as both their societal and institutional impacts.

We conclude with several recommendations for how policymakers, data stewards, and others can accelerate the use of private sector data to address data asymmetries and bolster FAIR and open science.

- First, data holding businesses should establish and empower data stewards to identify and act upon opportunities to support scientific advancement by providing functional access to private sector datasets. There will be no one-size-fits-all approach to this work. Businesses in different industries, subject to different laws and policies, and handling different types of data will need to take different pathways forward. Data stewards can be established as part of corporate social responsibility, research, and development, policy, or other departments or portfolios. Their role will entail collaborating responsibly to unlock data when there is a public interest case, protecting stakeholders by managing data ethically to prevent harm and misuse, and taking actions to ensure that insight is shared and translated into meaningful impact.
- Next, the scientific community should create a broadly recognized and validated system for monitoring and promoting uses and citations of private sector data to support research. Systems such as the Web of Science, ORCID, and Google Scholar can provide a useful overview of the influence and impact of research articles and papers. These systems serve dual purposes. First, they indicate what ideas, insights, and findings are guiding future research efforts and discoveries. Second, and perhaps equally important, they provide researchers with a credible record of the value and importance of their work. These citation tracking mechanisms can incentivize additional work and investment by researchers, their institutions, and their funders by demonstrating the reach and impact of data-sharing-driven research. A system that similarly captures and demonstrates the uptake, value, and impact of private sector contributions to scientific undertakings could likewise help to generate more activity and investment in increasing private sector data liquidity and addressing science data asymmetries. This may however require alternative means of citation and metrics of impact; and new means to acknowledge those that share data, not only the ones that generate insight from the data.

## Conclusion and avenues for further research

This article sought to fill some of the analytical gap regarding data asymmetries, and outline a set of possible solutions that could provide a toolbox for open science practitioners and data demand-side actors. We also examined how data holders and demand-side actors are experimenting with new and emerging operational models and governance frameworks for purpose-driven, cross-sector data collaboratives that bring to bear previously siloed datasets. These solutions were presented through a series of brief case studies on notable efforts to address science data asymmetries. We concluded with recommendations to unlock the public value of greater science data liquidity by empowering data stewards to provide functional access to private sector datasets and creating a broadly recognized and validated system for monitoring and promoting uses of private sector data to support research.

Moving forward, much more action and comparative research is needed to deepen our understanding toward:

- Increasing access to data (open data), including through:
  ° Descriptive and evaluative research to map and evaluate existing efforts to mitigate longstanding and deep-seated asymmetries in the data ecosystem, helping to develop new models of philanthropy and outreach to marginalized groups, and encouraging new technologies and institutional approaches that lower costs and increase access to data (e.g., architectural principles, zero-copy approaches, computational power redistribution, no-code tools, and data partnerships).
  ° Descriptive and evaluative research to explore whether new private-sector investments in data platforms and knowledge repositories are creating new types of data asymmetry or inequity.
  ° Descriptive and evaluative research on how data portability and interoperability are established and whether they actually impact the practice of data collaboration positively.
  ° Diagnostic research that can document the relationship and interplay between existing asymmetries stem and technological and societal drivers.
  ° Prescriptive analysis that can help reduce the capacity divide that limits the ability of certain groups and individuals to fully participate in the data economy, for example, through assessing what awareness-raising and educational efforts help increase data literacy and extend it beyond the narrow province of data scientists.
- Fostering a social license for data use (and reuse) toward the public good through:
  ° Descriptive research that can take stock of concerns over privacy and other violations that have increased public distrust over data and how it is used.
  ° Diagnostic analysis to assess how the resulting culture of risk aversion limits the potential contribution of data toward the public good.
  ° Diagnostic analysis and methodological development to measure the value of data reuse (i.e., secondary deployment of data originally collected for another purpose) toward public good applications.
  ° Prescriptive analysis that can provide pointers toward creating an ecology that enables responsible and trustworthy data use and reuse.
- Fostering public trust through:
  ° Descriptive and diagnostic analysis of new forms of participation, self-determination, and public discussion seeking to help engage and inform citizens—including citizen assemblies on the reuse of data for and in the public interest.
  ° Prescriptive research to prototype and experiment with new ways to provide agency beyond just consent).
  ° Diagnostic research on the impact of new models of data stewardship, as well as new professions and institutions, to unleash the potential of data in a trusted manner.
  ° Conceptual research to update existing notions of data ownership and that enable reuses of data for specific purposes.

## Author contributions

SV and AY contributed equally to the conception and drafting of the paper. Both authors contributed to manuscript revision, read, and approved the submitted version.

## Conflict of interest

The authors declare that the research was conducted in the absence of any commercial or financial relationships that could be construed as a potential conflict of interest.

## Publisher's note

All claims expressed in this article are solely those of the authors and do not necessarily represent those of their affiliated organizations, or those of the publisher, the editors and the reviewers. Any product that may be evaluated in this article, or claim that may be made by its manufacturer, is not guaranteed or endorsed by the publisher.

## Footnotes

^1^These and other over 200 other examples of private-sector data stewardship and cross-sector collaboration in the public interest are compiled and curated on the Data Collaboratives Explorer. Accessible at https://datacollaboratives.org/explorer.html/.
